# Preoperative Alignment and Interbody Cage Design Influence Radiographic Outcomes Following Anterior Cervical Discectomy and Fusion

**DOI:** 10.3390/jcm15031183

**Published:** 2026-02-03

**Authors:** Derrick Obiri-Yeboah, Zach Pennington, Hannah Levy, Abdelrahman Hamouda, Anthony L. Mikula, Kingsley Abode-Iyamah, Ian A. Buchanan, Chandan Krishna, Jeremy L. Fogelson, Benjamin D. Elder

**Affiliations:** 1 Department of Neurological Surgery, Mayo Clinic, Rochester, MN 55905, USA; obiriyeboah.derrick@mayo.edu (D.O.-Y.);; 2 Department of Orthopedic Surgery, Mayo Clinic, Rochester, MN 55905, USA; 3 Department of Neurological Surgery, Mayo Clinic, Jacksonville, FL 32224, USA; 4 Department of Neurological Surgery, Mayo Clinic, Phoenix, AZ 85054, USA

**Keywords:** ACDF, segmental correction, lordosis, interbody cage, cervical spine alignment

## Abstract

**Background**: Anterior cervical discectomy and fusion (ACDF) is a widely performed procedure for treating degenerative cervical spine conditions. While it effectively addresses neural decompression and restores segmental alignment, the interplay of baseline alignment and implant-specific factors on postoperative segmental alignment remains underexplored. This study evaluates the influence of preoperative cervical alignment and interbody cage design on segmental alignment changes following 1- to 3-level ACDF. **Methods**: Following institutional review board approval, we identified 258 patients undergoing ACDF for degenerative pathology between 1 January 2010 and 31 December 2023. Preoperative and postoperative radiographs were analyzed for cervical alignment, disc height, and segmental lordosis. Cage dimensions, lordosis, and positioning relative to vertebral landmarks were recorded. Multivariable linear regression models evaluated predictors of postoperative disc height, segmental lordosis, and their respective changes. **Results**: Postoperative disc height was positively associated with greater cage height (β = 1.13 mm per mm, *p* < 0.001) and negatively associated with greater cage lordosis (β = −0.10 mm per °, *p* = 0.001). Segmental lordosis was positively influenced by cage height (β = 0.78° per mm, *p* = 0.002) and lordosis (β = 0.42° per °, *p* = 0.002) but was negatively correlated with the distance of the cage from the anterior edge of the cranial vertebra (β = −1.76° per mm, *p* = 0.004). Greater preoperative segmental kyphosis predicted more significant postoperative lordosis correction (β = −1.07° per °, *p* < 0.001). **Conclusions**: This study underscores the importance of preoperative alignment and interbody cage design in achieving optimal segmental correction following ACDF. While cage height primarily drives disc height restoration, surgical technique, particularly anterior placement of the cage, is pivotal for enhancing segmental lordosis. These findings support personalized surgical planning to optimize alignment and patient outcomes.

## 1. Introduction

Annually, approximately 130,000 anterior cervical discectomy and fusions (ACDFs) are performed for cervical discogenic pathology [1]. It offers both direct spinal cord decompression through disc excision and indirect decompression through disc space height restoration, which also facilitates segmental alignment correction [2,3,4]. In this respect, ACDF can improve cervical sagittal alignment, and improved realignment may mitigate the risk of adjacent segment disease associated with subaxial spinal fusion [5].

Villavicencio and colleagues showed maintenance or improvement in sagittal alignment to correlate with superior patient-reported outcomes following ACDF [6]. Patients with preexisting cervical malalignment are at a greater risk of suboptimal results, underscoring the importance of restoring normal alignment [7]. Baseline alignment along with interbody cage characteristics and positioning—graft height, graft lordotic angle, and placement of the graft within the disc space—are key determinants of postoperative outcomes [6,7,8]. Taller and more lordotic cages can facilitate better sagittal balance restoration, while improper cage placement may limit the extent of realignment achievable [6,9,10].

Despite the established importance of these factors, few studies have evaluated the combined effects of baseline cervical alignment and cage design on segmental realignment after ACDF. This study aimed to assess how baseline alignment and surgical details interact to influence disc height and segmental lordosis restoration following 1- to 3-level ACDF.

## 2. Materials and Methods

Following institutional review board approval (22-013333) our data registry was queried to identify patients who underwent 1- to 3-level ACDF for degenerative cervical spine pathology prior to 31 December 2023. Given the retrospective nature of this study and the use of de-identified data, the requirement for individual patient consent was waived by the institutional review board. All patients were treated at a single institution by one of 24 surgeons. Inclusion criteria were preoperative and postoperative upright cervical radiographs suitable for alignment measurement, complete operative records detailing the interbody used, and ≥6 months of radiographic follow-up. Patients were excluded if they had a prior cervical fusion at treated levels, incomplete pre- or postoperative imaging, surgery for trauma, infection or neoplasm, or underwent a combined ACDF/cervical arthroplasty.

### 2.1. Data Collection

We recorded patient demographics (age, sex, and smoking status), surgical details (levels treated, cage height, cage lordosis, and cage material), and radiographic alignment parameters. Global alignment variables included C2–7 sagittal vertical axis (SVA), C2–7 lordosis, and T1 slope (T1S), all of which are routinely used in the assessment of cervical sagittal alignment in both clinical practice and research [11]. Segmental alignment parameters were disc height and segmental lordosis.

Disc height was measured at the anterior, middle, and posterior disc space, and averaged. Segmental lordosis was measured as the angle between the inferior endplate of the cranial vertebra and the superior endplate of the caudal vertebra at each treated segment. Measurements were obtained at three time points: preoperatively, immediate postoperative imaging (within 48 h), and the first upright radiographs obtained within 6 weeks. The primary postoperative radiographic endpoint for the multivariable models was the first upright radiograph obtained within 6 weeks. The primary postoperative radiographic endpoint for multivariable linear regression analyses was the first upright postoperative radiograph obtained within 6 weeks. All images were reviewed independently by two neurosurgery residents (DOY, ZP) with consensus reached in all cases. All radiographic measurements were performed on digital radiographs using the measurement tools within our institution’s PACS viewer.

### 2.2. Surgical Technique

Level and graft selection were determined by each individual surgeon, but all procedures employed a Smith–Robinson approach [12,13]. At our institution, the posterior longitudinal ligament is routinely resected, the uncovertebral joints are not, and the use of Caspar pins/intervertebral body distraction pins is surgeon-dependent.

### 2.3. Statistical Analysis

Data were recorded using Microsoft Excel (Redmond, WA, USA) and analyzed using SPSS Version 28.0.0. (IBM Corporation, Armonk, NY, USA). Primary outcomes of interest were change in average disc height, change in segmental lordosis, and segmental lordosis on the first postoperative upright radiograph. Secondary outcomes included loss of segmental correction and implant subsidence. Loss of segmental correction was defined as a ≥3° decrease in segmental lordosis on follow-up radiographs, selected as a pragmatic threshold intended to exceed typical measurement variability. Implant subsidence was defined as a ≥2 mm decrease in interspace height and severe subsidence as a ≥4 mm decrease. Interspace height was defined as the average of the anterior, middle, and posterior measurements between the inferior endplate of the cranial and superior endplate of the caudal level, consistent with prior radiographic methodology [14,15].

Continuous data are reported as median (IQR) and categorical data as counts (proportions). Multivariable linear regression was used to identify predictors of postoperative segmental lordosis and changes in segmental alignment, with β coefficients and 95% confidence intervals (CIs) reported. Analyses were performed at the treated level because cage morphology, cage position, disc height, and segmental lordosis are segment-specific. Variables of interest included preoperative alignment parameters, cage characteristics and patient demographics; predictors with a *p*-value < 0.05 were considered statistically significant.

For loss of correction and implant subsidence, Cox proportional hazards models were used for time-to-event analysis. For time-to-event analyses, time zero was defined as the date of surgery. Events were assessed on follow-up upright radiographs, and observations were censored at the date of the last available radiographic follow-up. Candidate variables were identified by univariable comparisons, considering demographics, radiographic parameters, cage dimensions, cage position, treated level, cage material, baseline bone quality (HU), and fixation type (integrated versus plate–interbody construct). Significant predictors on univariable analysis were entered into multivariable Cox regression using forward conditional with *p* < 0.05 for model entry and *p* > 0.10 for model removal.

## 3. Results

We identified 258 patients (median age 62.3 years [IQR 55.5, 68.4]; 50.7% male; Table 1) treated at 331 levels. The most common levels were C5/6 (34.6%), C4/5 (24.5%), and C6/7 (22.4%). Interbody material was titanium alloy in 78.2%, allografts in 20.0%, and polyetheretherketone (PEEK) in 0.6%. Anterior plate fixation was employed for 95.2% and integrated fixation cages in 3.6%. Median cage height was 7 mm [6.0, 8.0], lordosis 7° [6.0°, 10.0°], and cages were positioned a median 1.3 mm [0, 2.2] from the anterior edge of the cranial vertebra (Table 1).

Both disc height and segmental lordosis improved significantly. Median disc height increased by 3.7 mm [2.7, 4.6 mm], from 3.7 [2.9, 4.6 mm] preoperatively to 7.7 [6.6, 8.7 mm] postoperatively. Segmental lordosis increased by a median of 3.2° [0.1, 6.1°] from 3.5° [2.3°, 6.1°] to 7.6° [5.1°, 10.2°] (Table 1).

### 3.1. Predictors of Postoperative Disc Height and Disc Height Restoration

On multivariable regression (Table 2), postoperative disc height was predicted by greater cage height (β = 1.13 mm per mm, *p* < 0.001) while cage lordosis was negatively associated (β = −0.10 mm per °, *p* = 0.001 r^2^ = 0.84). Disc height restoration (Table 3) was likewise predicted by cage height (β = 1.12 mm per mm; *p* < 0.001), but negatively by higher preoperative disc height (β = −1.00 mm per mm, *p* < 0.001) and greater cage lordosis (β −0.10; *p* = 0.002, r^2^ = 0.94)

### 3.2. Predictors of Postoperative Segmental Lordosis and Change in Segmental Lordosis

Postoperative segmental lordosis (Table 4) was positively associated with cage height (β = 0.78° per mm, *p* = 0.002) and cage lordosis (β = 0.42° per °, *p* = 0.002), and negatively with distance from the anterior edge of the cranial endplate (β = −1.76° per mm; *p* = 0.004, r^2^ = 0.48). Change in segmental lordosis (Table 5) was predicted positively by cage height (β = 0.80° per mm; *p* = 0.002) and cage lordosis (β = 0.41° per °; *p* = 0.003) and negatively by greater preoperative segmental lordosis (β = −1.07° per °; [−1.24, −0.91]; *p* < 0.001) and greater distance of the interbody from the anterior edge of the cranial endplate (β = −1.84° per mm; *p* = 0.003, r^2^ = 0.57).

### 3.3. Predictors of Loss of Correction

Patients with ≥3° loss of segmental correction were older, had greater pre- and postoperative C2–7 lordosis, T1 slope, segmental lordosis and greater initial correction. On multivariable analysis (Table 6), predictors were higher preoperative C2–7 lordosis (HR 1.05 per °; *p* = 0.028) and higher postoperative segmental lordosis (HR = 1.25 per °; *p* <0.001).

### 3.4. Predictors of Subsidence

Patients with subsidence (≥2 mm) had greater postoperative disc height, lower postoperative segmental lordosis, more posterior cage placement, and narrower cages, though the latter two did not reach significance. On multivariable analysis, predictors were greater postoperative disc height (HR 1.39 per mm; *p* < 0.001) and smaller cage width (HR 0.79 per mm; *p* = 0.028) (Table 7). Subsidence ≥4 mm was too infrequent for analysis.

## 4. Discussion

Anterior cervical discectomy and fusion (ACDF) is widely used for degenerative cervical pathology, offering direct decompression and disc height restoration. Additionally, through anterior column lengthening, ACDF can improve segmental and global cervical alignment, though excessive distraction risks subsidence and worse outcomes. In our cohort (Table 1), the distribution of treated levels, implant types, and patient demographics reflected typical patterns of degenerative cervical disease, supporting the generalizability of our findings. In this study, disc height restoration was largely determined by implant size, whereas segmental lordosis correction depended on baseline alignment, cage properties, and particularly cage placement. Each millimetre closer to the anterior endplate corresponded to nearly 2° of additional segmental lordosis, highlighting placement as a critical factor. Implant choice dictated disc height restoration, but surgical technique was most important for sagittal correction.

### 4.1. Disc Height Restoration

Cage height was the most impactful predictor of both postoperative disc height and disc height restoration (Table 2 and Table 3) in our study, supporting the notion that taller cages primarily drive disc height restoration. Albert et al. reported a ~33% increase in neuroforaminal size after ACDF in 18 patients (25 levels) but found no correlation between graft size and foraminal height, cross-sectional area (CSA) enlargement, or symptom improvement [16]. Cho et al. studied 80 patients undergoing 1- to 3-level ACDF with PEEK or autograft and noted significant increases in mean foraminal height and CSA, although they did not analyze segmental correction or clinical outcomes [17]. Yang et al., in a cadaveric model, observed a parabolic relationship between graft height and foraminal size, with maximal area achieved at 160% of adjacent disc height [18].

In contrast, cage lordosis negatively correlated with postoperative disc height increase. Prior biomechanical work suggests that hyperlordotic cages may be difficult to seat in collapsed spaces, risking imperfect endplate apposition and reduced contact area, which predisposes to subsidence [19,20]. Fürderer et al. similarly found higher subsidence with cylindrical versus rectangular cages in bovine models [21]. Truumees et al. showed that larger grafts required greater distractive forces and endured higher compressive loads, leading them to recommend avoiding grafts >8 mm [22]. Hsueh et al. also reported that overdistraction increased subsidence and adjacent segment pathology, despite similar eventual correction and clinical improvement [23]. Collectively, these findings underscore the importance of balancing cage lordosis with achievable seating to avoid compromising height restoration or aggravating subsidence. Finally, shorter preoperative disc height predicted greater postoperative restoration, reflecting the greater correction potential in collapsed disc spaces.

### 4.2. Predictors of Change in Segmental Lordosis and Loss of Correction

Segmental lordosis correction was positively associated with cage height, cage lordosis, and more anterior cage placement (Table 4 and Table 5), while loss of correction was predicted by higher preoperative C2–7 lordosis and greater postoperative lordosis (Table 6). These findings support the role of taller and more lordotic cages in enhancing cervical alignment [24]. Although some studies, notably those of Kim et al. and Villavicencio et al., have reported no significant impact of lordotic versus flat implants in single-level ACDF [6,25], our results suggest that anterior placement within the disc space is the most important determinant of lordotic correction. This association has been well described in lumbar fusion, where anterior placement improves correction [5,26,27,28,29], but it remains sparsely studied in the cervical spine. Most cervical studies have focused on subsidence, noting that more anterior cage placement appears protective against subsidence, likely due to engagement of the anterior cortical rim, which has superior load-bearing properties relative to the centre of the disc space. Barsa and Suchomel noted patients experiencing subsidence to have a significantly greater space (2.59 mm vs. 0.82 mm) between the implant and anterior vertebral rim [20]. More recently, Jang et al. reported a three-fold lower subsidence rate in patients with implants placed within 2 mm of the anterior vertebral rim and greater segmental lordosis within the non-subsided group at 1 week postoperatively [30]. Similarly to disc height, less preoperative segmental lordosis predicted greater postoperative change. However, greater postoperative lordosis was linked with loss of correction over time, underscoring the need for balanced, not excessive, restoration.

### 4.3. Subsidence

Subsidence was predicted by use of a narrower cage and by greater postoperative disc height (Table 7). The strong positive association between postoperative disc height and subsidence risk underscores the importance of avoiding overdistraction during surgery. Prior biomechanical studies have demonstrated that graft oversizing increases the von Mises stresses on the vertebral endplates [31]. Excessive disc height restoration through placement of an oversized graft may exceed the physiological limits of the endplate, leading to implant settling, especially in patients with poor bone quality. Similarly, load sharing through a narrower interbody subjects the vertebral endplates to higher stresses, increasing the risk of implant subsidence and loss of correction. By contrast, the use of wider cages is protective, as larger implants provide greater surface area for load distribution and reduce stress on the endplate.

Implant material may also influence subsidence behaviour at the bone–implant interface. The prior comparative literature suggests differences in subsidence tendencies between titanium and PEEK cages, potentially related to differences in elastic modulus and endplate stress distribution, while newer cage designs and surface treatments may further affect osseointegration and settling [32,33].

### 4.4. Impact of Segmental Correction on Clinical Outcomes

Although the present study focuses on radiographic alignment outcomes and did not include patient-reported or functional measures, the prior literature has examined associations between disc height restoration, cervical alignment, and clinical recovery following ACDF. Interpretation of these studies depends in part on the outcome instruments used; for example, JOA/mJOA has recognized measurement limitations, including inter-rater variability, and should be interpreted with this in mind [34]. In a study of 57 patients treated with 1- or 2-level ACDF, Wu et al. reported that patients with implant subsidence had non-significantly worse recovery in Japanese Orthopedic Association (JOA) scores compared with those without subsidence, along with significantly lower disc height at last follow-up [35]. They also found that change in C2–7 lordosis correlated modestly with JOA recovery, suggesting a potential relationship between sagittal correction and clinical improvement. Hu and colleagues similarly reported that patients with restored alignment had better neck disability index (NDI) scores and greater improvement in neck pain following 1- or 2-level ACDF [36]. Other studies have shown mixed results: Wu et al. found no correlation between radiographic correction—measured by increased neuroforaminal height—and symptomatic improvement (JOA) after single-level ACDF, although greater disc space distraction was associated with greater improvement in segmental (but not global) alignment [37]. Hsueh et al. likewise reported no difference in JOA outcomes between patients with overdistraction (≥2 mm increase in disc height) and those treated with smaller implants [23]. Finally, Li et al. noted that maintenance of segmental correction was associated with superior clinical outcomes, whereas loss of segmental correction correlated with a negative trend in JOA scores over follow-up [38]. Taken together, these studies suggest that radiographic alignment and disc height restoration may be associated with clinical outcomes in some cohorts; however, clinical benefit cannot be inferred from radiographic changes alone and requires direct outcome measurement.

### 4.5. Limitations

This study focuses on early postoperative radiographic outcomes (immediate postoperative imaging and the first upright radiograph within 6 weeks). Patient-reported and functional outcomes were not analyzed; therefore, we cannot determine whether the observed radiographic changes translate into clinical benefit. Additionally, implant material comparisons are limited in our cohort because titanium/alloy cages comprised the vast majority of implants and PEEK cages were rare, precluding meaningful material-stratified inference. Because analyses were performed at the treated level and some patients underwent multilevel ACDF, within-patient correlation may violate independence assumptions and could underestimate standard errors; therefore, associations should be interpreted accordingly. Fusion status and pseudarthrosis were not systematically assessed with standardized CT imaging across the cohort; therefore, we cannot determine whether loss of correction occurred despite solid fusion versus in the setting of pseudarthrosis. Radiographic follow-up intervals were not standardized in this retrospective cohort, limiting our ability to report uniform follow-up duration metrics for the time-to-event analyses. Standardized longer-term radiographs (e.g., 6–12 months) were not consistently available across the cohort; therefore, we cannot determine whether early changes in disc height and segmental lordosis persist, attenuate, or converge over time. Future prospective studies with standardized long-term imaging are warranted.

## 5. Conclusions

In the present study, preoperative disc height, alignment, and interbody cage morphology were all predictive of postoperative segmental alignment. While cage morphometry dictates the majority of change in disc height, interbody placement within the disc space was the most important predictor of segmental lordosis correction. This suggests that implant selection may best determine disc height restoration, whereas sagittal plane correction depends heavily upon surgical technique. Further validation in prospective, multicenter cohorts is merited.

## Figures and Tables

**Table 1:** Summary of 331 treated levels.

Variable	n	Median [IQR]	n (%)
*Demographics*			
Age (yr)	331	62.3 [55.5, 68.4]	—
Sex (Male)	331	—	170 (50.7)
Race			
White		—	299 (89.3)
Black		—	16 (4.8)
Asian		—	4 (1.2)
Other		—	12 (3.6)
ASA	331	3 [2, 3]	—
*Preoperative Alignment*			
C2–7 SVA (cm)	258	2.98 [2.22, 4.42]	—
C2–7 Lordosis (°)	258	10.7 [5.9, 15.9]	—
T1 Slope (°)	256	33.0 [27.1, 37.0]	—
Segmental lordosis (°)	258	3.45 [2.30, 6.13]	—
Disc height (mm)	258		
Anterior		3.9 [3.0, 4.7]	—
Mid-disc		4.0 [3.1, 5.3]	—
Posterior		3.0 [2.3, 3.8]	—
Average		3.7 [2.9, 4.6]	—
*Surgical Details*			
Construct length	331	2 [1, 3]	—
Treated level	331		
C2/3		—	2 (0.6)
C3/4		—	49 (14.6)
C4/5		—	82 (24.5)
C5/6		—	116 (34.6)
C6/7		—	75 (22.4)
C7/T1		—	7 (2.1)
Cage Material			
Ti/Ti Alloy		—	262 (78.2)
Allograft		—	67 (20.0)
PEEK		—	2 (0.6)
Fixation			
Plate		—	319 (95.2)
Integrated fixation cage		—	12 (3.6)
Cage Height (mm)	331	7 [6, 8]	—
Cage Length (mm)	314	12.5 [12.0, 14.0]	—
Cage Width (mm)	313	15.0 [14.0, 16.0]	—
Cage Lordosis	331	7.0 [6.0, 10.0]	—
Cage Position	255		
Anterior edge cranial level (mm)		1.3 [0, 2.2]	—
Anterior edge caudal level (mm)		1.5 [0.7, 2.8]	—
Posterior edge cranial level (mm)		4.9 [2.9. 6.7]	—
Posterior edge caudal level (mm)		4.2 [2.8, 6.3]	—
*First Postop Radiograph*			
C2–7 SVA (cm)	258	3.32 [2.36, 4.41]	—
C2–7 Lordosis (°)	255	14.1 [8.9, 23.1]	—
T1 Slope (°)	254	34.9 [29.1, 39.5]	—
Segmental lordosis (°)	258	7.6 [5.1, 10.2]	
Disc height (mm)	258		
Anterior		8.6 [7.4, 9.7]	—
Mid-disc		8.3 [7.0, 9.2]	—
Posterior		6.2 [5.4, 7.3]	—
Average		7.7 [6.6, 8.7]	—
Δ Segmental lordosis	199	3.2 [0.1, 6.1]	—
Δ average disc height	198	3.7 [2.7, 4.6]	—

Key: ASA—American Society of Anesthesiologists; PEEK—polyetheretherketone; SVA—sagittal vertical axis; yr—year.

**Table 2:** Multivariable analysis for predictors of disc height on first postoperative radiograph.

Variable	β	95% CI	*p*
Cage height (per mm)	1.13	[1.01, 1.25]	<0.001
Cage lordosis (per °)	−0.10	[−0.17, −0.04]	0.001

Key: mm—millimetre. Model explains 84.3% of variance in data (adjusted r^2^ = 0.843).

**Table 3:** Multivariable analysis for predictors of change in disc height on first postoperative radiograph.

Variable	β	95% CI	*p*
Preoperative average disc height (per mm)	−1.00	[−1.04, −0.96]	<0.001
Cage height (per mm)	1.12	[1.00, 1.24]	<0.001
Cage lordosis (per °)	−0.10	[−0.17, −0.04]	0.002

Key: mm—millimetre. Model explains 93.5% of variance in data (adjusted r^2^ = 0.935).

**Table 4:** Multivariable analysis for predictors of segmental lordosis on first postoperative radiograph.

Variable	β	95% CI	*p*
Cage height (per mm)	0.78	[0.28, 1.28]	0.002
Cage lordosis (per °)	0.42	[0.16, 0.68]	0.002
Distance to anterior edge of cranial level (per mm)	−1.76	[−2.94, −0.57]	0.004

Key: mm—millimetre. β coefficients are reported in degrees (°) of segmental lordosis per unit increase in the predictor. Model explains 48.3% of variance in data (adjusted r^2^ = 0.483).

**Table 5:** Multivariable analysis for predictors of change in segmental lordosis on first postoperative radiograph.

Variable	β	95% CI	*p*
Preoperative segmental lordosis (per °)	−1.07	[−1.24, −0.91]	<0.001
Cage height (per mm)	0.80	[0.30, 1.30]	0.002
Cage lordosis (per °)	0.41	[0.14, 0.67]	0.003
Distance to anterior edge of cranial level (per mm)	−1.84	[−3.05, −0.64]	0.003

Key: mm—millimetre. β coefficients are reported in degrees (°) of change in segmental lordosis per unit increase in the predictor. Model explains 57.2% of variance in data (adjusted r^2^ = 0.572).

**Table 6:** Survival analysis for predictors of loss of segmental correction (≥3° decrease in segmental correction).

Variable	HR	95% CI	*p*-Value
SL on 1st upright X-ray (per °)	1.25	[1.12, 1.40]	<0.001
Preop C2–7 lordosis (per °)	1.05	[1.01, 1.10]	0.028

Key: SL—segmental lordosis.

**Table 7:** Survival analysis for predictors of implant subsidence (≥2 mm height loss).

Variable	HR	95% CI	*p*-Value
Cage width (per mm)	0.79	[0.64, 0.98]	0.028
Postoperative disc height (per mm)	1.39	[1.20, 1.61]	<0.001

Key: mm—millimetre.

## Data Availability

All de-identified data can be made available upon request.
